# Severity, extent, distribution and predisposing factors of gingival recession in Turkish patients: a cross-sectional study

**DOI:** 10.4317/medoral.26956

**Published:** 2025-03-23

**Authors:** Halil Çelik, Hatice Selin Güngörmek

**Affiliations:** 1 Department of Oral and Dental Health, Vocational School of Health Care Services, Istinye University, Istanbul, Türkiye; 2 Department of Periodontology, Faculty of Dentistry, Marmara University, Istanbul, Türkiye

## Abstract

**Background:**

The aim of this cross-sectional study was to assess the extent, severity, distribution and potential predisposing factors of gingival recession (GR), utilizing a questionnaire and clinical periodontal measurements obtained from Turkish patients.

**Material and Methods:**

A total of 534 subjects were examined. Participants meeting the inclusion criteria evaluated by dental hygiene habits, educational level, smoking habit and past orthodontic treatment. Plaque index (PI), gingival index (GI), probing depth (PD), bleeding on probing (BOP), clinical attachment level (CAL), gingival thickness (GT), high frenum attachment, and mobility were recorded on the tooth with GR. Probe transparency (PT), crown width/crown length ratio (CW/CL), papilla height (PH) and height of gingival scallop were measured on the index tooth (#11FDI). The GR severity was categorized by using Miller’s classification.

**Results:**

Of the 534 individuals examined in this study, 376 (70.4%) had gingival recession, while 262 patients (49%) were meeting the inclusion criteria and 2,721 teeth (37%) were affected. The majority of the teeth (44.8%) showed Miller class I. The highest GR frequency was detected in incisors (39.5%), particularly in mandible. The correlation between GR and PI (*p*=0.025), PD (*p*=0.034), PH (*p*=0.007), CW/CL (*p*=0.009), CAL (*p*<0,001), PT (*p*<0,001) was found statistically significant. No statistical relation was found between tooth brushing duration (*p*>0,05), tooth brushing frequency (*p*>0,05) and gingival recession.

**Conclusions:**

Gingival recession is a multifactorial condition significantly influenced by clinical and anatomical parameters such as PI, PH, CW/CL, PT while toothbrushing habits, including duration and frequency, appear to have a minimal impact.

** Key words:**Gingiva, gingival recession, epidemiology, etiology, phenotype.

## Introduction

Gingival recession (GR) is the apical shift of the gingival margin towards the cemento-enamel junction (CEJ). Although the etiology of GRs is not fully understood, it has been reported that they present a multifactorial nature (1). Risk factors associated with GR are categorized into three groups as anatomical, physiological and pathological. Anatomical factors include alveolar dehiscence, high frenulum attachment, tooth position, gingival morphology and phenotype (2,3) while physiological risk factors are age, gender, genetic predisposition and orthodontic tooth movement (4,5). Pathological risk factors can be listed as periodontal diseases, occlusal trauma, periodontal treatment failures, improper restorative and surgical procedures, smoking, traumatic oral hygiene habits and piercings (5,6).

GR is a common mucogingival problem; its prevalence presents considerable differences among population, ranging from 41-90% in adults depending on the methods of analysis, population, and age group examined (2,7). No consensus exists on the frequency of GR by gender (1,8). A number of studies reported that the incidence and severity of GR increase with age (2,9,10).

Epidemiological studies are crucial for understanding the distribution and determinants of health conditions in specific populations, providing insights into risk factors, informing preventive strategies, and guiding public health policies. The Turkish study included 831 participants (537 females and 294 males) aged 15-68 years and reported an overall prevalence of GR at 78.2%, with 76% in females and 82% in males. Key contributing factors identified were traumatic toothbrushing, frequency of toothbrushing, high frenulum attachment, inadequate oral hygiene, dental plaque, and dental calculus (2). Understanding the prevalence and etiology of GR in a given population is necessary for its control and prevention, and hence, the present study aimed at assessing the severity, extent and potential predisposing factors of GR via a questionnaire and clinical parameters in Turkish patients.

## Material and Methods

- Study design

This is a single-center cross-sectional study. The study protocol was approved by the Marmara University Faculty of Dentistry Clinical Research Ethics Committee (22.02.2018/2018-170). The individuals who agreed to participate in the study were given detailed information about the study and informed consent forms were obtained in accordance with the 1975 Helsinki Declaration as revised in 2013.

- Study population

The subjects were selected from the patient population who applied to the Periodontology Clinic of Dental Faculty, Marmara University in Istanbul, Turkey. The enrollment of the individuals in this study was based on the following; systemically healthy, non-pregnant (for female subjects), between the ages of 18-65 years and presented at least one buccal side GR at any single or multiple teeth. In addition, the participants should have tooth #11 FDI without any crown restoration or filling that served as the index tooth. Patients who met the inclusion criteria and agreed to participate in the study were divided into two groups according to gender.

The study population consisted of all patients who visited our clinic during the specified period. The sample size was calculated based on data from a similar study (2), using the Instat* program. Power analysis, assuming a gingival recession prevalence of 78.2%, a 95% confidence interval, and a 5% margin of error, determined that the minimum required sample size was 262 patients.

- Questionnaire

The patients were interviewed with a questionnaire investigating their demographic data, oral hygiene habits including daily toothbrushing frequency, toothbrush type (manual or electric), toothbrush bristle hardness (hard, medium, soft), toothbrushing duration, dental history, smoking habit, clenching and/or grinding habits and past orthodontic treatment.

- Clinical examination

Full mouth periodontal clinical parameters including plaque index (PI) to determine the quantity of plaque on the tooth surface using 0-3 score (score 0: no plaque; score 1: a film of plaque adhering to the gingival margin and adjacent surfaces of the tooth which may be seen by using periodontal probe; score 2: moderate accumulation of plaque within the gingival pocket, tooth surfaces and gingival margin which may be seen with the naked eye; score 3: abundance of plaque within the gingival pocket, tooth surfaces and gingival margin); gingival index (GI) to assess the severity of gingival inflammation with a score from 0 to 3 (score 0: normal gingiva; score 1: mild inflammation, slight change in colour, slight edema, no bleeding on probing; score 2: moderate inflammation, redness, edema, and bleeding on probing; score 3: severe inflammation, marked redness and edema, ulceration tendency to spontaneous bleeding); probing depth (PD), the distance from the gingival margin to the bottom of the gingival sulcus; bleeding on probing (BOP) scored (0 absent; 1 present) within 30 s after probing gingival pocket; and clinical attachment level (CAL), the distance from the CEJ to the bottom of the gingival sulcus were recorded in six surfaces per tooth.

**Figure 1:**
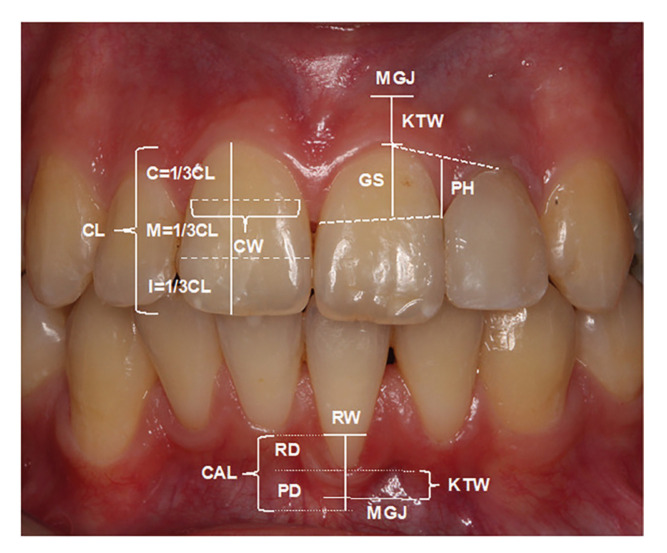
Measurement of clinical parameters. C: Cervical, CAL: Clinical attachment level, CL: Crown length, CW: Crown width, GS: Height of gingival scallop, I: Incisal, KTW: Keratinized tissue width, M: Middle, MGJ: Mucogingival junction, PD: Probing depth, PH: Papilla heigth, RD: Recession depth, RW: Recession width.

As shown in Fig. 1, additional measurements for tooth presenting GR in patient including recession depth (RD), distance from CEJ to the apical extent of gingival margin; recession width (RW), horizontal distance from distal to the mesial margin of recession at CEJ level; keratinized tissue width (KTW), distance from the gingival margin to the mucogingival junction; gingival thickness (GT), the distance between the tip and silicon stopper measured by a digital caliper on #20 endodontic spreader that is placed perpendicular to the gingiva under local anesthesia at the buccal aspect of recession 3 mm apically from gingival margin; presence of high frenulum attachment recorded as present (1) or not (0); and mobility assessed according to mobility score (score 0: physiological mobility; score 1: increased mobility but less than 1 mm; score 2: mobility more than 1 mm; score 3: more than 1 mm displacement combined with a displacement in vertical direction) were recorded.

GRs severity were classified according to Miller’s (11) and Cairo (12) classification. Panoramic radiograph and periapical X-rays of the relevant teeth were assessed in conjunction with the intraoral findings, using the GR classification to evaluate interdental bone loss. The extent of GR was evaluted based on the percentage of affected teeth and classified as localized (less than 15% of teeth) or generalized (15% or more of teeth) (13).

In order to define gingival phenotype of each individual, probe transparency (PT) was assessed by placing the probe into labial sulcus of the index tooth and recorded as a categorical variable [yes: probe visible (thin gingiva); no: probe not visible (thick gingiva)] (14). As shown in Fig. 1, in order to calculate crown width/crown length ratio (CW/CL), at first crown length of the index tooth was measured as the distance from the incisal edge to the buccal gingival margin followed by dividing this distance into three equal portions as cervical, middle and incisal; then the crown width was recorded as the distance between approximal crown surfaces at the borderline between the middle and cervical portions (15). A line was drawn to connect the most apical points of the gingival margin of teeth #11 FDI and #12 FDI, and the distance from the top of the papilla to this line was recorded as the papilla height (PH) (Fig. 1) (15). Height of gingival scallop (GS) was measured as the distance from the line formed by the connection of the peaks of two adjacent interdental papillae to the most apical position of the buccal marginal gingiva (Fig. 1) (14). KTW and GT were also recorded from the index tooth.

All clinical measurements were performed by a calibrated examiner (HC) using a periodontal probe (University of North Carolina Probe, Hu-Friedy, Chicago, USA). Before the initiation of the study, the calibration was carried out in 10 non-study subjects with GR, and RD measurement was repeated twice with 1 week interval. The reproducibility of the examiner resulted in an intraclass correlation coefficient of 0.836 (95%CI, 0.764-0.873) for the RD measurement.

- Statistical analysis

All analyses were carried out using STATA® (Stata Corp LLC, USA) 15.1 version. Kolmogorov-Smirnov test was used for normality. The clinical parameters were presented as minimum-maximum, percentage, mean, and standard deviation. Mann Whitney-U test and Pearson Chi-square test were used for comparisons of quantitative and qualitative variables between groups, respectively. Spearman correlation analysis, backward selection method and multinominal logistic regression analysis were applied in order to model the relationship between GR and independent variables. Confidence interval was taken as 95% (α = 0.05), *p*<0.05 was considered as statistically significant.

## Results

In this study, out of the 534 individuals examined, a total of 376 subjects (70.4%) presented GR. However, 262 patients met the inclusion criteria and agreed voluntarily to participate in the study. The patients’ demographic variables are shown in Table 1. The mean age of all subjects was 43.43±10.55 years, of which 53% were female (*n*=139, mean age 40,21±11,03 years) and 47% were male (*n*=123, mean age 45,22±10,46 years). The majority of the individuals (43%, *n* = 113) were graduated from primary school. While 66% (*n*=174) of the individuals participating in the study were non-smoker, smoking habit of males was significantly higher than females (*p*<0.05). Majority of the population were using manual (94%) toothbrush with medium type of bristles (60.3%) and brushing their teeth for 2 minutes (37.8%) 2 times a day (46.2%). The mean value of toothbrushing duration was 1.67±0.89 min. In addition, 42.0% of total subjects had tooth clenching or grinding habits without any significant difference between genders (*p*>0.05). Also, 6.1% of subjects, mostly females (9.4%), had undergone orthodontic treatment (*p*<0.05). The patients’ full mouth clinical variables presented no differences between the female and male patients, as shown in Table 1.

The mean RD was 2.14±0.65 mm in all patients and with the highest incidence of GR (70.4%) ranging between 1-2 mm. The severity of GR was found to be significantly higher in females than males (*p*<0.05). When GR was categorized according to the Miller’s classification, 44.8% of GR presented Miller class I; 26.1% Miller class II, 19.3% Miller class III, and 9.8% Miller class IV. When GR was categorized according to the Cairo’s classification, 70.9% of GR presented RTI; 19.3% RTII and 9.8% RTIII. There were no statistically significant differences between female and male patients in terms of RD, RW, KTW, GT and mobility values of teeth presenting gingival recessions (*p*>0.05).

**Figure 2:**
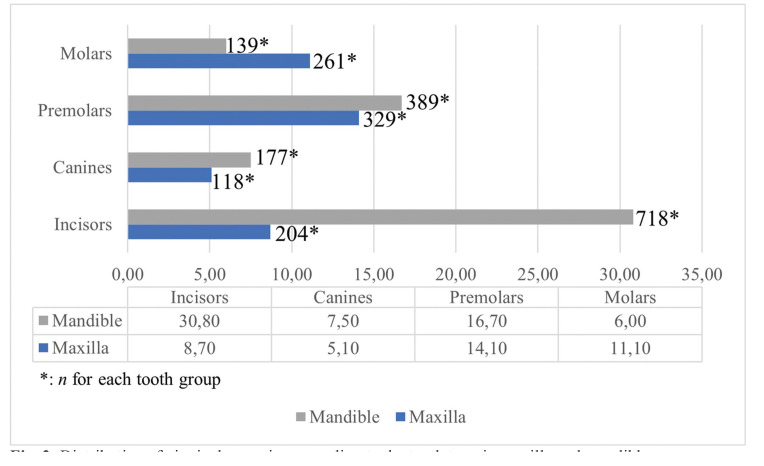
Distribution of gingival recession according to the tooth type in maxilla and mandible.

Parallel to the finding of higher PT in female patients compared to males (*p*<0.05), GT was found to be significantly thinner (*p*<0.001) (Table 1). Fig. 2 shows distribution of GR according to the tooth type in maxilla and mandible.

**Figure 3:**
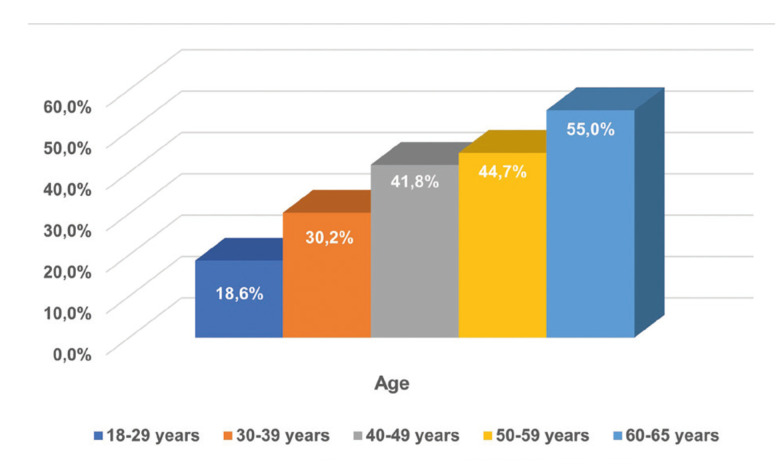
Distribution of prevalence of gingival recession according to age.

The highest GR frequency was detected in incisors (39.5%), particularly in mandible, followed by premolars (30.8%), molars (17.1%) and canines (12.6%). Moreover, the prevalence of GR was found to increase with increasing age (Fig. 3).

Correlations between the prevalence of GR and demographic/clinical variables are shown in Table 2. While positive correlations were found between the prevalence of GR at tooth level and full mouth PI (*p*<0.05), PD (*p*<0.05), CAL (*p*<0.05) and PT (*p*<0.01) of the index tooth.

The negative correlations were present between the prevalence of GR at tooth level and KTW (*p*<0.01), CW/CL (*p*<0.01) and PH (*p*<0.01) of the index tooth.

Table 3 and Table 4 show the results of multivariate linear regression for each factor associated with the extent and severity of recessions. The multivariate analysis reported that age (OR= 1.14; 95% CI 1.06-1.23; *p* = 0.001), clenching and grinding habits ( OR= 0.25; 95% CI 0.08-0.82; *p*= 0.022), PD (OR= 2.52; 95% CI 0.00- 0.83; *p* = 0.037), CAL (OR= 27.95; 95% CI 2.41- 324.59; *p*= 0.008), RW (OR=1.95; 95% CI 1.07-3.56; *p*=0.029) and GT (OR=0.26; 95% CI 0.09-0.72; *p*= 0.009) were significantly associated with extent of GR. In the multivariate regression model, age (OR= 1.04; 95% CI 1.01-1.07; *p*= 0.008), CAL (OR=3.05; 95% CI 1.67-5.57; *p*=0.000), PT (OR= 2.28; 95% CI 1.17-4.42; *p*=0.015) and CW/CL (OR= 2.00; 95% CI 1.86-4.29; *p*=0.019) were associated with severity of GR.

## Discussion

In this cross-sectional study, severity, extent, distribution and potential causative factors for severity and extent of GR were evaluated in healthy subjects via a specially designed questionnaire and clinical parameters.

In the present study, the prevalence of GR at patients’ level was detected as 70.4% in 534 subjects, which is consistent with the reported prevalence of GR varying from 58.5 to 99.7% in other large population cross sectional studies (4,15-17).

GRs severity recession depth (> 5 mm) was only observed in 3.8% of teeth, while 26.0% were 3-4 mm depth and most of the sample was characterized by shallow GRs with approximately 70.2% of the affected teeth presenting a 1-2 mm recession. The findings of the present sample are consistent with results from epidemiological studies from adult populations with both low and high levels of oral hygiene (16,18,19).

The distribution of recessions in our study patients was detected higher on the mandibular incisors. Our findings are in agreement with that reported by Toker *et al*. (2), Albandar *et al*. (4), Rios *et al*. (16). In contrast with other studies detected a higher prevalence in the maxilla (5,6), including the cuspids, premolars or molars the most affected tooth. Differences in age, oral hygiene habits, anatomy may in part explain the discrepancies within the results.

Frequency of GR among young people (15-25 years) was found to be 26.9% while among middle-aged individuals (45-60 years) was 70.27% (10). In our study, 52.7% of the subjects were female and 47.3% were male; however, the prevalence of GR did not differ between genders and there was no relationship between GR and gender. In another study, the prevalence of GR was stated to be 72.5% in all individuals examined and was higher in female (75%) than male (70%) (3). No consensus has been reached in the studies evaluating the association between GR and gender.

In this study, since it is considered that education level may change tooth brushing habits, education level was a parameter that was questioned. Similarly with our findings, Chrysanthakopoulos *et al*. (20) was reported that no significant association between education level and GR was found.

Smoking is considered a potential factor that exacerbates gingival recession (GR) (2,10). In the present study, 33.6% (*n*=88) of the participants were smokers, but no significant association was observed between smoking and GR. In contrast to our findings, Mythri *et al*.. (10) reported a smoking prevalence of 7.1% and proposed that smoking could be a predisposing factor for GR. Conversely, other studies have suggested that smoking may not be a significant risk factor for the development and the extent of GR (20,21).

In the present study, it was observed that the majority of patients used manual toothbrushes with medium bristles. However, no significant relationship was found between the severity and extent of gingival recession (GR) and toothbrush type (*p* > 0.05). This finding contrasts with studies that have suggested an association between toothbrush type, bristle hardness, brushing duration, and frequency with GR (18,20,22). Consistent with the current study's results, Kallestal & Uhlin (23) found no significant correlation between GR and toothbrushing factors. They also reported that 25% of participants brushed their teeth more than once a day and noted no gender differences in oral hygiene habits.

In the present study, PI and BOP values were slightly higher in males without any significance. Additionally, positive correlation was detected a between GR and PI. Similarly with these findings, studies revealed that the rate of GR had a relationship with PI, presence of calculus, and BOP (10,19-22).

Even though a definitive conclusion is unclear, gingival phenotype is another causative indicator in development of GR (24). In this study, GT of an index tooth was found to be thinner in female (1.19 mm) compared to male (1.36 mm). In 82% (*n*=200) of the subjects, GT of an index tooth was found to be >1 mm. However, no association was found between the GR and GT. Olsson *et al*. (15) found that higher percentage of GR in subject levels were associated with the thin gingival phenotype. On the other hand, Shah *et al*. (25) stated that no significant relationship was detected between GT, age and gender. Another study indicates that thin gingival phenotype (42.4%) was less common among female compared to male (55.6%) (26). In another study, there was a negative relationship between GT and PT (14). Evaluating relationship between GT and genders, it was found to be thicker in male (27). Likewise, Muller *et al*. (28) found that GT was significantly thinner in female.

In some studies, high FA is thought to play a role in GR (2,3). In the present study, high FA was seen in 3.4% of the teeth existing GR (*n*=91), there was no significant relationship between high FA and severity of GR. One of the epidemiological studies investigating the risk factors GR in Turkish population stated that the overall prevalence of high FA was 14.2%, in the regions with GR, this rate was 4.2% (2). Similar to our study, Nguyen-Hieu *et al*. (3) found the incidence of high FA in teeth with GR as 8% and stated that the relationship between high FA and GR was not statistically significant.

In our study, periodontal probe could not be seen in only 22.5% of the subjects. De Rouck *et al*. (27) found that 57% of the individuals they examined, gingival phenotype was thick and periodontal probe could not be seen. The reason for this may be that all our study group consists of individuals with thinner gingival phenotype. Moreover, in our study, statistically significant negative relationship was found between the GR and PT.

Another clinical measurement evaluated in this study; CW/CL was calculated on index tooth based on the study of Olsson *et al*. (15). In the present study it was observed that there was a negative relationship between the prevalence of GR and CW/CL . In this study, CW/CL rate was found to be 0.78. De Rouck *et al*. (27) determined this rate as an average of 0.81 in 100 patients. This ratio is thought to be an effective parameter on the gingival phenotype. Stein *et al*. (14) found a significant positive correlation between GT and CW/CL. According to a recent study conducted by Liu *et al*. (24) no significant relationship was detected between GT and CW/CL.

When the severity of the GR is measured, in most studies, including our study, Miller’s class I GR is observed most frequently (10,19). This is usually followed by Miller’s class II, III and IV GR (10).

In our study, GR was most frequently encountered in the mandibular anterior teeth (30.8%), and least in the mandibular molars 5.9%. Similar to the present study, Mythri *et al*. (10) stated that mandibular incisors were the most affected teeth group by GR (43%). GR in the mandibular incisors was mainly associated with inadequate oral hygiene (29), and GR in the premolars mainly caused by traumatic tooth brushing (7). There are various opinions about GR seen in molars. While some studies related traumatic tooth brushing to GR in the maxillary 1st molar (30), there were also findings indicating that dental plaque and calculus were the primary etiological factor in these teeth (19). It has been reported that GR were frequently encountered in anterior region of the mandibular and posterior region of the maxilla (9).

In our study, incidence of GR in Turkish adult population was found to be 70.4%. Since 7 out of 10 patients examined by clinicians may have GR in Turkey, more effort should be put forth by the clinicians to educate the patients about indicators for gingival recession, and appropriate protective oral hygiene practices to decrease the occurrence of GR.

## Conclusions

The present cross-sectional study demonstrated a high prevalence of buccal gingival recessions in a sample of Turkish patients who applied to the periodontology clinic in a dental faculty. The factors associated with the extent of GR were age, clenching and grinding habits, probing depth, clinical attachment level, and factors associated with severity of GR were age, probe transparency and crown width /crown length ratio. However, no relationship was found with tooth brushing habits.

## Figures and Tables

**Table 1:** Demographic characteristics and full-mouth clinical variables of patients categorized by gender.

Variable (Mean ± SD (Min/Max)		Total n=262	Female n=139	Male n=123	p*
Age		43.43 ± 10.55 (21.00/65.00)	40.21 ± 11.03 (21.00/63.00)	45.22 ± 10.46 (21.00/65.00)	0.101
Education level	Primary school	113 (43.1)	59 (42.4)	54 (43.9)	0.936
	High school	75 (28.5)	42 (30.2)	33 (26.8)	0.772
	University	61 (23.5)	31 (22.3)	30 (24.4)	0.984
	Post-graduate	13 (4.9)	7 (5.0)	6 (4.9)	0.973
Smoking	Smoker	88 (33.6)	32 (23.0)	56 (45.5)	0.000
	Non-smoker	174 (66.4)	107 (77.0)	67 (54.5)	0.032
Type of toothbrush	Manual	248 (94.6)	132 (95.5)	115 (93.5)	0.871
	Electric	14 (5.4)	7 (6.5)	8 (6.5)	0.943
Toothbrush bristle hardness	Hard	24 (9.0)	18 (12.9)	6 (4.8)	0.602
	Medium	165 (63.0)	81 (58.4)	84 (68.4)	0.901
	Soft	73 (28.0)	40 (28.7)	33 (26.8)	0.701
Toothbrushing duration	<1 min	29 (9.1)	13 (9.4)	16 (8.9)	0.797
	1 min	87 (33.2)	50 (36.0)	37 (30.9)	0.842
	2 min	98 (37.4)	49 (35.3)	49 (40.7)	0.855
	>2 min	48 (18.3)	27 (19.4)	21 (19.5)	0.905
Toothbrushing frequency	Never	9 (3.5)	4 (2.9)	5 (4.1)	0.873
	1 time a day	119 (45.4)	49 (35.2)	70 (56.9)	0.003
	2 times a day	121 (46.1)	77 (55.4)	44 (35.7)	0.003
	>2 times a day	13 (5.0)	9 (6.5)	4 (3.3)	0.024
Tooth clenching or grinding habits	Yes	152 (58.1)	79 (56.8)	73 (59.3)	0.681
	No	110 (41.9)	60 (43.2)	50 (40.7)	0.654
Orthodontic treatment	Yes	16 (6.1)	13 (9.4)	3 (2.4)	0.021
	No	246 (93.9)	126 (90.6)	120 (97.6)	0.708
Toothbrushing duration (min)		1.67 ± 0.89 (0.00/3.00)	1.65 ± 0.89 (0.00/3.00)	1.72 ± 0.88 (0.00/3.00)	0.619
Full mouth clinical variables	Plaque index	1.24 ± 0.41 (0.23/2.43)	1.22 ± 0.45 (0.23/2.12)	1.26 ± 0.48 (0.25/2.43)	0.124
	Gingival index	1.19 ± 0.42 (0.00/2.50)	1.19 ± 0.49 (0.20/2.20)	1.18 ± 0.41 (0.00/2.50)	0.565
	Bleeding on probing (%)	28.50 ± 11.36 (0.00/67.00)	28.30 ± 11.40 (0.00/67.00)	28.70 ± 11.20 (0.00/67.00)	0.279
	Probing depth (mm)	2.31 ± 0.48 (0.80/3.60)	2.33 ± 0.45 (1.50/3.60)	2.32 ± 0.41 (1.43/3.24)	0.947
	Clinical attachment level (mm)	3.19 ± 0.77 (1.56/6.30)	3.16 ± 0.74 (1.74/5.02)	3.23 ± 0.80 (1.56/6.30)	0.441
Clinical variables of teeth presenting GR	Frenulum attachment (n of teeth)	91 (3.3)	48 (2.9)	43 (3.6)	0.950
Recession depth	1-2 mm	1910 (70.2)	989 (66.7)	921 (74.3)	0.002
	3-4 mm	569 (20.9)	387 (26.1)	182 (14.7)	0.027
	≥5 mm	242 (8.9)	105 (7.2)	137 (11.0)	0.068
Miller	Class I	1219 (44.8)	629 (42.5)	590 (47.6)	0.064
	Class II	711 (26.1)	455 (30.7)	256 (20.6)	0.627
	Class III	525 (19.3)	322 (21.7)	203 (16.4)	0.925
	Class IV	266 (9.8)	75 (5.1)	191 (15.4)	0.322
RT	RT1	1929 (70.9)	1008 (68.1)	921 (84.3)	0.064
	RT2	526 (19.3)	272 (18.4)	254 (20.5)	0.925
	RT3	266 (9.8)	201 (13.5)	65 (5.2)	0.322
Recession depth (mm)		2.14±0.65 (1.00/5.00)	2.18±0.67 (1.00/5.00)	2.10±0.62 (1.00/3.75)	0.534
Recession width (mm)		3.06±1.00 (0.00/6.75)	3.04±1.03 (0.00/6.75)	3.06±0.98 (0.00/6.75)	0.461
Keratinized tissue width (mm)		5.43±4.56 (1.67/7.00)	5.39±4.55 (1.67/6.00)	5.47±4.59 (1.67/7.00)	0.475
Gingival thickness (mm)		1.65±1.12 (0.27/6.70)	1.62±1.12 (0.27/6.70)	1.08±0.29 (0.31/6.70)	0.295
Mobility		0.22±0.74 (0.00/4.00)	0.27±0.84 (0.00/4.00)	0.17±0.59 (0.00/3.00)	0.358
Probe transparency	Yes	203 (77.5)	115 (43.9)	88 (33.6)	0.017
	No	59 (22.5)	23 (8.77)	36 (13.7)	-
Keratinized tissue width (mm)		4.49±1.03 (3.00/7.00)	4.56±1.08 (3.00/7.00)	4.42±0.98 (3.00/7.00)	0.320
Gingival thickness (mm)		1.27±0.30 (0.68/1.98)	1.19±0.25 (0.68/1.90)	1.36±0.32 (0.90/1.98)	0.000
Papilla heigth(mm)		4.72±0.72 (3.00/6.00)	4.78±0.68 (3.00/6.00)	4.66±0.76 (3.00/6.00)	0.195
Crown width/crown length		0.77±0.09 (0.58/1.00)	0.77±0.08 (0.58/1.00)	0.78±0.08 (0.58/1.00)	0.482
Height of gingival scallop (mm)		3.41±1.07 (1.00/5.00)	3.48±1.06 (1.00/5.00)	3.33±1.08 (1.00/5.00)	0.220

*Pearson Chi-Square test, ^**^Mann Whitney-U test, *p*<0.05.

**Table 2:** Correlations between the prevalence of gingival recession at teeth level and demographic/clinical variables.

Variables	Prevalence of Gingival Recession	
	rho	p*
Age	0.062	0.317
Gender	-0.068	0.269
Education	0.063	0.308
Smoking habit	0.065	0.296
Toothbrush type	0.065	0.295
Toothbrush bristle hardness	-0.026	0.677
Toothbrushing frequency	-0.061	0.327
Toothbrushing duration	0.070	0.256
Clenching and grinding habits	0.050	0.417
History of orthodontic treatment	0.012	0.848
Frenulum attachment	0.057	0.361
Plaque index	0.138	0.025
Gingival index	-0.088	0.157
Probing depth (mm)	0.135	0.034
Bleeding on probing (%)	-0.015	0.811
Clinical attachment level (mm)	0.727	0.000
Keratinized tissue width (#11) (mm)	-0.194	0.002
Gingival thickness (#11) (mm)	-0.018	0.771
Probe transparency (#11)	0.299	0.000
Crown width/Crown length (#11)	-0.160	0.009
Gingival scallop height (#11) (mm)	0.000	1.000
Papilla height (#11) (mm)	-0.165	0.007
Keratinized tissue width	-0,616	0.000
Gingival thickness	-0.526	0.000
Recession depth	0.271	0.000
Recession width	0.194	0.002

Spearman correlation test, p*<0.05.

**Table 3:** Final multivariate linear regression (stepwise model) for extent of gingival recession as dependent variables.

Extent of gingival recession	Variable				
	Beta	SE	OR	95% CI	p*
Age	0.131	0.038	1.14	1.06-1.23	0.001
Clenching and grinding habits	-1.378	0.602	0.25	0.08-0.82	0.022
Plaque index	0.923	0.748	2.52	0.58-10.90	0.217
Probing depth	-3.142	1.507	0.04	0.00-0.83	0.037
Clinical attachment level	3.330	1.251	27.95	2.41-324.59	0.008
Recession depth	-0.711	0.445	0.49	0.20-1.17	0.110
Recession width	0.669	0.307	1.95	1.07-3.56	0.029
Keratinized tissue width	-0.084	0.182	0.92	0.64-1.31	0.645
Gingival thickness	-1.334	0.513	0.26	0.09-0.72	0.009

SE: Standart error, OR: Odds ratio, 95% CI: 95% confidence interval, p*<0.05.

**Table 4:** Final multivariate linear regression (stepwise model) for severity of gingival recession as dependent variables.

Severity of gingival recession	Variable				
	Beta	SE	OR	95% CI	*p*
Age	0.038	0.014	1.04	1.01-1.07	0.008
Gender	-0.500	0.320	0.60	0.33-1.10	0.102
Plaque index	0.448	0.336	1.56	0.81-3.02	0.182
Clinical attachment level	1.115	0.308	3.05	1.67-5.57	0.000
Probe transparency (#11)	0.824	0.338	2.28	1.17-4.42	0.015
Crown width/Crown length (#11)	3.862	1.653	2.00	1.86-4.29	0.019
Gingival scallop height (#11)	-0.132	0.133	0.88	0.67-1.14	0.322

SE: Standart error, OR: Odds ratio, 95% CI: 95% confidence interval, *p*<0.05.

## Data Availability

The datasets used and/or analysed during the current study are available from the Correspondence on reasonable request due to privacy reasons and large data size.
